# Vitamin A Supplementation Induces AMFK Production to Promote Cartilage Proliferation and Antler Growth in Sika Deer

**DOI:** 10.3390/ani15192879

**Published:** 2025-10-01

**Authors:** Huazhe Si, Songze Li, Huanhuan Liu, Xing Duan, Ruijia Deng, Yuhang Zhu, Yunxi Zhang, Sibo Chen, Shaoying Wang, Cuiliu Ma, Yongxiang Li, Jianan Sang, Xiuhua Gao, Hanlu Liu, Weixiao Nan, Zhipeng Li

**Affiliations:** 1 College of Animal Science and Technology, Jilin Agricultural University, Changchun 130118, China; sihuazhe1989@163.com (H.S.); ze961672411@163.com (S.L.); lhhz5590@163.com (H.L.); dx010726@163.com (X.D.); drj020409@163.com (R.D.); yuhangzhu199512@163.com (Y.Z.); zyun_xi@163.com (Y.Z.); q1059019684@163.com (S.C.); winnewang@126.com (S.W.); x735416612@163.com (C.M.); yongxiang9831@163.com (Y.L.); sjn1996918@outlook.com (J.S.); xiuhuagao@126.com (X.G.); zhplicaas@163.com (Z.L.); 2 College of Agriculture, Chifeng University, Chifeng 024000, China; 3 Joint International Research Laboratory of Modern Agricultural Technology, Ministry of Education, Jilin Agricultural University, Changchun 130118, China; 4 Jilin Provincial Engineering Research Center for Efficient Breeding and Product Development of Sika Deer, Jilin Agricultural University, Changchun 130118, China; 5 Key Laboratory of Animal Production, Product Quality and Security, Ministry of Education, Jilin Agricultural University, Changchun 130118, China

**Keywords:** antler transcriptomics, fecal microbiota, Sika deer, vitamin A

## Abstract

Antlers are unique mammalian organs that grow rapidly through extensive cartilage proliferation. Vitamin A (VA), via retinoic acid, is known to influence skeletal development, yet its contribution to antler growth remains unclear. We investigated whether rumen-protected VA supplementation modulates antler biology in sika deer by integrating gut microbiota profiling, fecal and serum metabolomics, antler cartilage transcriptomics, and in vitro chondrocyte assays. VA was associated with compositional shifts in the gut microbiota and altered metabolic profiles in feces and serum. Notably, the melatonin-derived metabolite AMFK increased in both matrices and showed strong associations with antler weight and cartilage-related gene expression. VA-supplemented deer exhibited higher antler weight, and antler transcriptomes indicated changes in pathways consistent with cartilage extracellular matrix and growth signaling. In chondrocytes, AMFK promoted proliferation and upregulated chondrogenic markers. Together, these findings suggest that VA may promote antler growth through a microbiota–metabolite–gene axis rather than by altering systemic retinol alone.

## Simple Summary

Sika deer antlers grow rapidly and renew each year. We explored whether vitamin A (VA) supplementation could support antler growth by interacting with the gut microbiota and the small molecules they produce. Using measurements from deer and laboratory tests on cartilage cells, we found signals suggesting that VA may influence antler biology through microbiota-related metabolites. These findings are preliminary and based on a small number of animals, but they point to a potential nutritional route to improve antler production that deserves further study.

## 1. Introduction

Antlers are the only known mammalian organs capable of complete periodic regeneration, driven by rapid cartilage proliferation followed by bone formation [1,2]. This remarkable feature makes it an excellent model for studying cartilage development and bone formation in mammals [3]. Antlers can grow up to 2.75 cm per day and mineralize by 2.5 to 3.0 g calcium per day [2,4]. Although endocrine and nutritional factors regulate antler growth [5], the availability of nutrients, particularly proteins, vitamins, and minerals, is a key determinant of the growth rate [6,7,8]. Among these nutrients, the fat-soluble vitamin A (VA) is of special interest for its role in regulating chondrocyte proliferation [9]. Recent studies have shown that retinoic acid (RA), the active VA metabolite, can restore the regenerative potential in mammals by reactivating genetic programs that were silenced during evolution [10]. This suggests that VA may facilitate rapid antler growth by promoting chondrocyte differentiation. However, the mechanisms by which VA regulates antler growth remain poorly understood. Elucidating these mechanisms may uncover key metabolites and signaling pathways that support cartilage proliferation and antler development.

Retinoic acid binds retinoic acid receptors and retinoid X receptors, forming heterodimers that interact with retinoic acid response elements to regulate transcriptional programs involved in cell differentiation, apoptosis, and development [11]. During cartilage development, RA typically reduces the early expansion of undifferentiated mesenchymal progenitor cells, promotes their commitment to the chondrogenic lineage, and supports chondrocyte proliferation [12]. The cartilage of sika deer (*Cervus nippon*) antlers is characterized by rapid chondrocyte proliferation and high expression of key cartilage-related genes such as *SOX9* and *COL2A1*. Since VA has been shown to upregulate the expression of these genes in cartilage [13], it may promote antler growth by modulating their expression and the associated chondrocyte functions.

Recent studies have shown that dietary VA can reshape the structure and function of the gut microbiota. Vitamin A may influence the abundance of other functionally important microbial taxa, including Clostridiales and Ruminococcaceae [14], which are increasingly recognized for their roles in host lipid metabolism and the production of bioactive metabolites involved in endocrine and signaling pathways [15,16]. In addition to modulating microbial communities, VA has been shown to profoundly affect both intestinal and systemic host metabolism [14]. Furthermore, VA may alter the biosynthesis and circulation of key metabolic hormones, such as melatonin [17]. As a pleiotropic signaling molecule, melatonin regulates the circadian rhythm, antioxidative defense, and tissue regeneration, and has been implicated in antler growth by promoting chondrocyte proliferation, stimulating angiogenesis, and inhibiting osteoclast activity [18,19]. Of particular interest is acetyl-N-formyl-5-methoxykynurenamine (AMFK), a melatonin-derived metabolite with potential activity in cartilage biology. Therefore, we hypothesize that vitamin A supplementation will alter the gut microbiota and its metabolite profile, increasing the production of AMFK, which in turn correlates with antler cartilage gene expression and antler weight.

In this study, we aimed to investigate whether rumen-protected vitamin A (VA) supplementation in sika deer (i) reshapes fecal bacterial community features and the fecal metabolite profiles; (ii) alters serum metabolite profiles, with particular attention to the melatonin-derived metabolite; and (iii) increases the antler weight and influences gene transcription in antler cartilage.

## 2. Materials and Methods

### 2.1. Animals and Experiment Design

Twelve healthy four-year-old male sika deer with similar body weights (107.6 ± 2.87 Kg) and antler casting time (around 15 June) were selected for this study. The animals were randomly assigned to two groups: the control (Con, *n* = 6) group, which received a standard basal total mixed ration, and the VA group (VA, *n* = 6), supplemented with 10 g/day of rumen-protected VA (40,000 IU/day, in the basal diet (Supplementary Table S1). Rumen-protected VA (50% VA content) was provided by King Techina (Hangzhou, China). Because deer-specific daily requirements during antlerogenesis are not established, we used a short-term, fixed dose of rumen-protected VA (≈40,000 IU/day) to provide a clear mechanistic contrast, guided by evidence that retinoids are locally present and synthesized in growing antlers and that local RA signaling supports antler development [7]. For safety, animals were monitored daily for appetite, demeanor, and locomotion; serum biochemistry and serum retinol were assessed at study end, and no adverse clinical signs were observed. All animals were housed in individual pens measuring (4 × 5 m). Environmental enrichment was provided through fixed scratching posts and salt-mineral licks, with periodic rotation, and pens included shade structures, dry-bedded resting areas, and a sand patch for dusting. Animals had ad libitum access to fresh water and received a standard total mixed ration, fed twice daily at 07:00 a.m. and 04:00 p.m. each day. The trial lasted 8 weeks (15 June 2023 to 15 August 2023), including a 1-week adaptation period. Husbandry and all procedures complied with institutional and national animal-welfare guidelines and were approved by the Animal Ethics Committee of Jilin Agricultural University (protocol 20230314017; 14 March 2023).

### 2.2. Sample Collection and Measurement

At the end of the experiment, antlers were harvested and weighed, and the cartilage samples were collected for transcriptomic analysis. Before antler harvesting, animals were anesthetized with xylazine (1.0–1.5 mg/kg, intramuscular [IM]) followed by ketamine (2–4 mg/kg, IM). After clipping and aseptic preparation of the antler base, a tourniquet was applied around the pedicle to minimize blood loss. The antler was transected just above the pedicle coronet (approximately 2 cm above the pedicle) using a sterile fine-toothed saw, taking care to avoid pedicle injury. Hemostasis was confirmed before the gradual release of the tourniquet; the stump was then treated with a topical antiseptic and protected according to welfare guidelines. Immediately after removal, antlers were weighed, and distal cartilage tissue was dissected under sterile conditions, snap-frozen in liquid nitrogen, and stored at −80 °C until RNA extraction. Following sample collection, atipamezole (0.1–0.2 mg/kg, IM) was administered to reverse the effects of xylazine. Fresh feces were obtained by rectal retrieval under gentle manual restraint using single-use lubricated obstetrical sleeves and sterile gloves; approximately 5–10 g was collected into pre-labeled sterile tubes and immediately stored in liquid nitrogen for 16S rRNA sequences and untargeted metabolomic analysis. Blood samples were obtained via jugular venipuncture into heparinized evacuated tubes, followed by centrifugation at 3500× *g* for 15 min at 4 °C to isolate serum for untargeted metabolomic analysis.

### 2.3. DNA and RNA Extraction and Sequencing

Microbial DNA was extracted from feces using the QIAamp Fast DNA Stool Mini Kit (51604, QIAGEN, Hilden, Germany) according to the manufacturer’s protocol. Total RNA was extracted from approximately 100 mg of antler cartilage tissue using TRIzol reagent (15596026CN, Invitrogen, Carlsbad, CA, USA) following the manufacturer’s instructions. DNA and RNA concentrations were assessed using a BioTek Epoch2 (BioTek Instruments, Winooski, VT, USA), and RNA integrity was evaluated with an Agilent 2100 Bioanalyzer (Agilent Technologies, Santa Clara, CA, USA). For 16S rRNA gene sequencing, the V3–V4 hypervariable regions of the bacterial 16S rRNA gene were amplified using primers 341F (5′-CCTAYGGGRBGCASCAG-3′) and 806R (5′-GGACTACNNGGGTATCTAAT-3′). PCR products were purified with AMPure XP beads (A63881, Beckman Coulter, Brea, CA, USA) and quantified using a Qubit 3.0 fluorometer (Thermo Fisher Scientific, Waltham, MA, USA). Sequencing libraries were prepared with Illumina TruSeq DNA PCR-Free Library Prep Kit (Illumina Inc., San Diego, CA, USA) and sequenced on an Illumina MiSeq platform (2 × 300 bp paired-end reads). For bulk RNA sequencing, RNA (1 μg) with an integrity number (RIN) ≥ 7.0 was used for library construction. Poly(A)+ mRNA was isolated using oligo(dT) magnetic beads and fragmented into fragments (~200–300 bp). First-strand cDNA synthesis was performed with random hexamer primers, followed by second-strand synthesis. The resulting double-stranded cDNA underwent end repair, A-tailing, and adaptor ligation. Libraries were then amplified and purified using AMPure XP beads (A63882, Beckman Coulter, Brea, CA, USA). Paired-end sequencing (2 × 150 bp) was performed on the Illumina HiSeq 4000 platform.

### 2.4. Untargeted Metabolome of Feces and Serum and Analysis

Feces and serum were processed using methanol-based extraction protocols optimized for LC-MS analysis [20]. Approximately 20 mg of frozen feces was thawed on ice and extracted with 400 μL of methanol/water (7:3, *v*/*v*) containing internal standards. After vortexing and centrifugation, the supernatant was collected for subsequent metabolomic analysis. For serum, 50 μL of thawed serum was mixed with 200 μL of precooled methanol (−20 °C), vortexed, and subjected to three freeze–thaw cycles to enhance metabolite release. The mixture was then centrifuged at 13,000× *g* for 10 min at 4 °C, and the supernatant was collected. Chromatographic separation was performed on a Waters ACQUITY Premier HSS T3 column (186009469, 2.1 × 100 mm, 1.8 μm; Waters Corporation, Milford, MA, USA) maintained at 40 °C. The mobile phase consisted of solvent A (0.1% formic acid in water) and solvent B (0.1% formic acid in acetonitrile), with the following gradient: 5–20% B over 1 min, increased to 99% B over the next 2 min, held for 1.5 min, and then returned to 5% B for re-equilibration. The flow rate was 0.4 mL/min, and the injection volume was 4 μL. Mass spectrometric detection was carried out on a Q Exactive™ Orbitrap system operated in both positive and negative ion modes under standard electrospray ionization conditions. Raw data were processed and analyzed using the MetaboAnalyst 6.0 [21]. Principal component analysis (PCA) was performed to evaluate global variation between groups. Differential metabolites were identified using the Mann–Whitney U test with a threshold of *p* < 0.05, followed by Kyoto Encyclopedia of Genes and Genomes (KEGG)-based pathway enrichment analysis. Source attribution of key metabolites (host-, microbiota-, co-metabolism-derived) was conducted using MetOrigin (2.0) [22]. Serum metabolites were further analyzed with random forest analysis using the randomForest package (v4.7) to identify the top-ranking metabolites contributing to group separation.

### 2.5. Bioinformatics Analysis

Raw sequences of the 16S rRNA gene were analyzed using QIIME 2 [23]. After quality control and denoising via the DADA2 algorithm, amplicon sequence variants (ASVs) were identified [24]. Taxonomic classification was assigned based on the SILVA 138 database [25]. The sequencing data were rarefied to the minimum sequencing depth across all samples to normalize ASV counts. Alpha diversity indices (including ACE, Chao1, and observed ASVs) and beta diversity metrics (Bray–Curtis dissimilarity and unweighted UniFrac distances) were computed using the Microeco (v0.7.1) package [26]. Alpha diversity indices were compared by Mann–Whitney. Principal coordinates analysis (PCoA) was conducted to assess microbial community structure. Group differences were statistically evaluated using PERMANOVA (999 permutations). Functional predictions of microbial communities were performed using the Tax4Fun2 package (v1.1.5), which infers KEGG Orthology (KO) functional profiles. The predicted KO abundance matrix was used for subsequent pathway enrichment and visualization [27].

For transcriptome analysis, RNA-seq reads were mapped to the sika deer genome (NCBI Assembly: PRJNA831044) using HISAT2 (v2.2.1) [28], and gene expression levels were quantified with StringTie (v2.2.1) [29]. DESeq2 was applied to identify differentially expressed genes (DEG) between Con and VA groups (v3.21) [30], using a threshold of |log_2_ fold change| > 1.5 and false discovery rate (FDR) < 0.05. KEGG pathway enrichment was conducted using the clusterProfiler package (v4.6.0). Correlation networks were visualized using igraph (v1.5.1) and ggraph (v2.1.0) based on DEGs significantly correlated (*p* < 0.05) with either *DHRS3* or *CYP26B1*. Linear regression analysis was conducted between normalized gene expression levels and antler weight using the car package (v3.1). Additionally, protein–protein interaction (PPI) analysis was performed using the STRING database (v12.0, https://string-db.org (accessed on 26 September 2025)), with a minimum required interaction score of 0.15. Weighted gene co-expression network analysis (WGCNA) was performed using DEGs to identify gene modules associated with antler weight and key differentially abundant metabolites [31].

Spearman correlation analysis was performed between serum metabolites and antler weight using the ggplot2 package (v4.0.0). Correlations with an absolute coefficient (|r|) > than 0.5 and a *p*-value < 0.05 were considered statistically significant. The resulting metabolite–microbiota interaction network was constructed and visualized using the igraph (v1.5.1) package.

### 2.6. Isolation and Culture of Antler Chondrocytes

Antler chondrocytes (ACCs) were isolated from antler cartilage as previously described with minor modifications [32]. Briefly, cartilage tissue at the antler tip was carefully dissected free of perichondrium and soft tissues, minced into ~1 × 1 mm pieces, and rinsed thoroughly in sterile HBSS/DMEM to remove blood and debris. Cartilage was first digested in 0.25% trypsin-EDTA (HY-K3007, MCE, Monmouth Junction, NJ, USA) for 30 min at 37 °C with gentle agitation. The suspension was pelleted (~1000 rpm, ~200× *g*, 5 min), and the tissue was then digested in 0.25% collagenase II (17101015, Gibco, Billings, MT, USA) in PBS for 2 h at 37 °C. The digest was passed through a 125 μm sterile nylon mesh to remove undigested fragments, and cells were collected by centrifugation (~200× *g*, 10 min) and washed twice in HBSS. Viable cells were counted by trypan blue exclusion and seeded at 2 × 10^5^ cells per well/dish in DMEM (C11995500BT, Gibco, Billings, MT, USA) supplemented with 10% FBS (F8318, Sigma, St. Louis, MO, USA), 100 U/mL penicillin and 100 μg/mL streptomycin, and cultured at 37 °C, 5% CO_2_. The medium was changed every 2–3 days.

### 2.7. Cell Proliferation Assays

For the Cell Counting Kit-8 (CCK-8) assays, ACCs were seeded in 96-well plates at 2000 cells/well. After 48 h of treatment with AMFK (0, 0.1, 1, or 10 μM), 10 μL of CCK-8 solution was added to each well and incubated at 37 °C for 1.5 h. Absorbance at 450 nm was measured using a BioTek Epoch2 microplate reader (BioTek Instruments, Winooski, VT, USA). EdU staining was performed using the BeyoClick™ EdU Cell Proliferation Kit with Alexa Fluor 555 (C0075S, Beyotime, Shanghai, China), following the manufacturer’s instructions. Briefly, ACCs were cultured in a 24-well plate at 2000 cells/well for 48 h. Then, 10 μM EdU was added to each well, and the plate was incubated at 37 °C for 2 h. After fixation and permeabilization, nuclei were stained with 0.5 mL Hoechst 33342 for 10 min at room temperature in the dark. Images were captured using a CKX53 microscope (Olympus, Tokyo, Japan). The proportion of EdU-positive cells was quantified by counting cells in five randomly selected fields per well.

### 2.8. Immunofluorescence Assay

ACCs were seeded on glass coverslips in 24-well plates and treated with AMFK (0 or 1 μM) for 48 h. Cells were fixed with 4% paraformaldehyde (P0099, Beyotime, Shanghai, China) and permeabilized with 0.1% TritonX-100 (P0096, Beyotime, Shanghai, China). The cover glass was then rinsed and blocked in 5% BSA (AR0004, Boster, Wuhan, China) for 10 min. After washing with PBS, the coverslips were incubated overnight at 4 °C with primary antibodies: anti-COL2A1 (43306, Cell Signaling Technology, Danvers, MA, USA, 1:1000) and anti-SOX9 (82630, Cell Signaling Technology, Danvers, MA, USA, 1:1000). The slides were then incubated with the Alexa Fluor 488-conjugated secondary antibodies (SA00003-2, Proteintech, Wuhan, China) for 45 min, followed by nucleus staining with DAPI (4′6-diamidino-2-phenylindole, H-1200-10, Vectorlabs, Newark, CA, USA) for 5 min for nucleus labeling. Fluorescent images were captured using an Olympus microscope.

### 2.9. Cell RNA Extraction and qPCR

When the chondrocytes reached approximately 80% confluence, they were harvested, and the total RNA was extracted using TRIzol reagent (15596026CN, Invitrogen, Carlsbad, CA, USA). Complementary DNA (cDNA) was generated from 1 μg of total RNA using the TransScript^®^ Uni All-in-One First-Strand cDNA Synthesis SuperMix for Quantitative real-time PCR (qPCR, TransGen Biotech, Beijing, China), according to the manufacturer’s instructions. qPCR was performed on the qTOWER^3^G system (Analytik, Jena, Germany) using *PerfectStart*^®^ Green qPCR SuperMix (TransGen Biotech, Beijing, China). Glyceraldehyde-3-phosphate dehydrogenase (GAPDH) served as the endogenous control. Relative transcript levels were determined using the 2^−ΔΔCt^ method. Primer sequences are listed in Supplementary Table S2, and all primers were synthesized by Sangon Biotech (Shanghai, China).

### 2.10. Statistical Analysis

Prespecified primary outcomes (antler weight and antler average daily gain) were analyzed using Mann–Whitney U tests. For families of parallel comparisons (e.g., the serum biochemistry panel and α-diversity indices), we controlled the false discovery rate (FDR) using the Benjamini–Hochberg procedure and report adjusted *p* values (significance threshold < 0.05). Procedures for multi-feature omics analyses (microbiome, metabolomics, RNA-seq), including their FDR control, are described in Section 2.5 Bioinformatics Analysis.

## 3. Results

### 3.1. VA Supplementation Alters Gut Microbiota Composition

**Figure 1:**
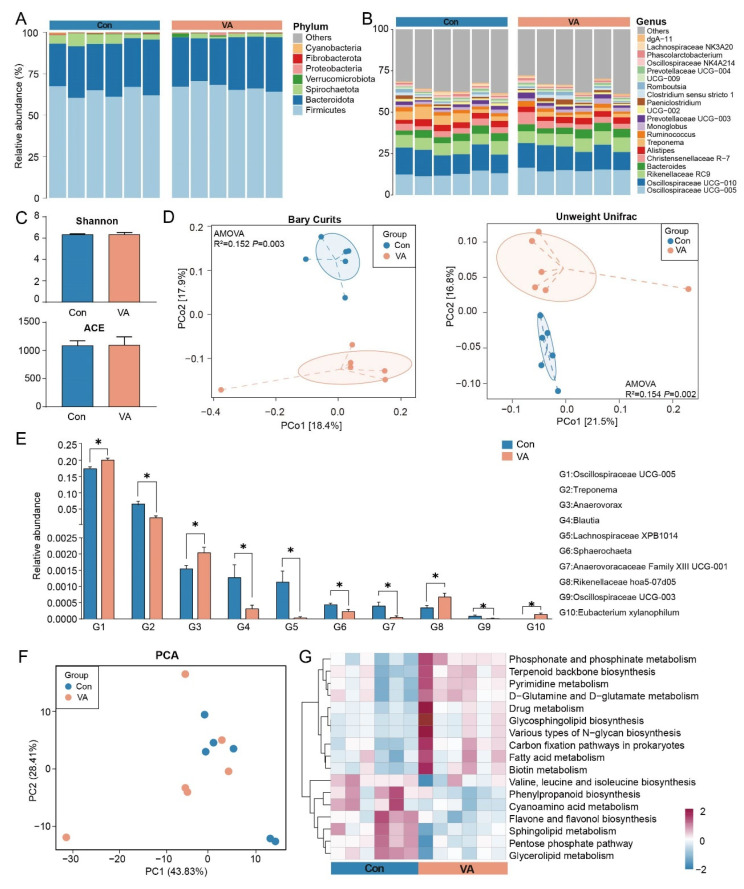
**Effects of vitamin A supplementation on fecal microbiota composition and predicted functional profiles in sika deer:** (**A**) Relative abundance of bacterial phyla in fecal samples. (**B**) Relative abundance of bacterial genera in fecal samples. (**C**) Alpha diversity indices (Shannon and ACE) of bacterial communities. (**D**) Principal coordinates analysis (PCoA) plots based on Bray–Curtis dissimilarity and unweighted UniFrac distance matrices. (**E**) Bar plot of differential bacterial genera between groups, * *p* < 0.05. (**F**) Principal component analysis (PCA) of predicted metabolic pathways at KEGG level 3. (**G**) Heatmap of predicted functional pathways inferred from 16S rRNA gene data using Tax4Fun2.

We first examined the fecal bacteria of the sika deer based on 16S rRNA gene sequences, and on average, 72,875 high-quality reads were obtained for each sample (63,246 to 80,311). A total of 1662 ASVs were obtained across all of the samples, which were classified into 11 phyla. Firmicutes (Con: 63.8%, VA: 66.9%), Bacteroidota (Con: 29.9%, VA: 29.8%), and Spirochaetota (Con: 4.9%, VA: 1.81%) were the predominant phyla (Figure 1A). At the genus level, Oscillospiraceae UCG-005 (Con: 12.6%, VA: 15.0%), Oscillospiraceae UCG-010 (Con: 11.4%, VA: 9.4%), Rikenellaceae RC9 (Con: 7.4%, VA: 7.5%), Bacteroides (Con: 4.2%, VA: 4.4%), and Christensenellaceae R7 (Con: 3.5%, VA: 4.0%) were both predominant in both groups (Figure 1B). Alpha diversity indices did not differ significantly between Con and VA (Figure 1C). PCoA indicated a group-associated pattern supported by PERMANOVA (Bray–Curtis: R^2^ = 0.152, *p* = 0.003; unweighted UniFrac: R^2^ = 0.154, *p* = 0.002; Figure 1D), consistent with compositional rather than within-sample diversity differences. Moreover, the relative abundances of Oscillospiraceae UCG-005, *Anaerovorax*, the Rikenellaceae gut group, and the *Eubacterium xylanophilum* group were significantly higher in the VA group than those in the Con group (*p* < 0.05, Figure 1E). In contrast, the relative abundances of *Treponema*, *Blautia*, Lachnospiraceae unclassified, Sphaerochaeta, Anaerovoracaceae Family XIII UCG-001, and Oscillospiraceae UCG-003 were significantly higher in the Con group than those in the VA group (*p* < 0.05, Figure 1E).

We also applied TaxFun2 to predict the potential metabolic functions of the fecal microbiota, and a PCA based on KEGG level 3 metabolism pathways showed overlaps between the two groups (Figure 1F). Moreover, the relative abundances of 10 metabolic pathways, including fatty acid metabolism and phosphonate and phosphinate metabolism, as well as valine, leucine, and isoleucine biosynthesis; phenylpropanoid biosynthesis; and cyanoamino acid metabolism, were significantly higher in the VA group compared to those in the Con group (*p* < 0.05, Figure 1G). Conversely, seven metabolic pathways were significantly more abundant in the Con group than in the VA group.

### 3.2. Effects of VA Supplementation on Fecal Metabolites

**Figure 2:**
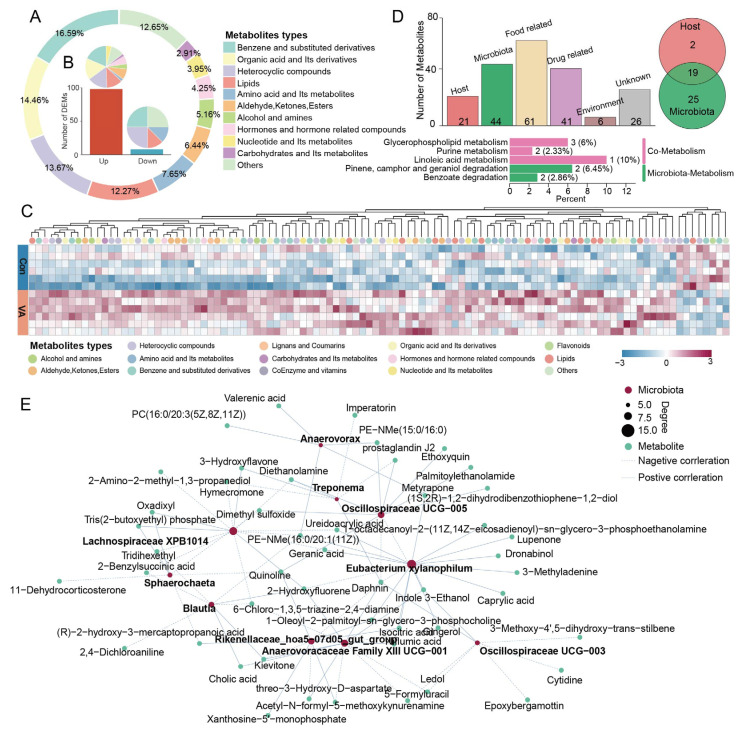
**Fecal metabolite profiles and metabolite-microbiota associations in sika deer following vitamin A supplementation:** (**A**) Pie chart of metabolite categories detected in fecal samples. (**B**) Number and classification of up- and down-regulated differential metabolites. (**C**) Heatmap of fecal metabolite profiles classified by compound types. (**D**) Source attribution and KEGG pathway enrichment analysis of increased metabolites. (**E**) Correlation network between differential fecal metabolites and bacterial genera.

To investigate VA supplementation’s metabolic consequences in the gut, we performed untargeted metabolomic analyses of feces. A total of 2545 metabolites were identified in both the Con and VA groups and classified into 11 categories, with the most abundant classes being benzene and substituted derivatives (16.59%), organic acids and their derivatives (13.67%), heterocyclic compounds (12.27%), lipids (12.88%), and amino acids and their metabolites (7.65%; Figure 2A). Compared to the Con group, 98 metabolites were significantly increased, and 8 were significantly decreased in the VA group (Figure 2B,C, Supplementary Table S3). The increased metabolites were predominantly from benzene derivatives (e.g., vanillic acid, curcumin, and secoisolariciresinol), organic acids (e.g., 5-dehydro-4-deoxy-D-glucaric acid, valerenic acid, and isocitric acid), heterocyclic compounds (e.g., rutaecarpine and quinoline), and lipids (e.g., 2-Benzylsuccinic acid and 16-Methylheptadecanoic acid). In addition, six hormones and hormone-related compounds, including 15-Deoxy-delta-12,14-prostaglandin J2, poststerone, 11-Dehydrocorticosterone, prostaglandin J2, epoprostenol, and norepinephrine, were increased, suggesting that hormone metabolism in the gut was enhanced by VA supplementation. To understand the potential contributions of the gut microbiota and host to the altered metabolic landscape induced by VA, we performed metabolite origin tracing analyses using MetOrigin (Supplementary Table S4). Of the 98 increased metabolites, 44 were predicted to be derived from microbial metabolism, 21 from the host, and 19 from co-metabolism involving both microbial and host metabolism (Figure 2D). The KEGG pathway enrichment analysis showed that microbially derived metabolites were primarily enriched in the degradation of pinene, camphor, and geraniol degradation and benzoate. Meanwhile, 19 metabolites derived from co-metabolism were enriched in the glycerophospholipid metabolism, purine metabolism, and linoleic acid metabolism pathways, whereas the host-derived metabolites did not enhance any KEGG pathways.

To further investigate the associations between the differential genera and fecal metabolites, a correlation network analysis was conducted. The genus Oscillospiraceae UCG-005 showed significant positive correlations with prostaglandin J2, palmitoylethanolamide, and diethanolamine, and negative correlations with daphnin and 3-Hydroxyflavone (Figure 2E). *Anaerovorax* was positively correlated with epoprostenol and 1-Oleoyl-2-palmitoyl-sn-glycero-3-phosphocholine. Similarly, the genus *Eubacterium xylanophilum* also exhibited a positive correlation with 1-Oleoyl-2-palmitoyl-sn-glycero-3-phosphocholine. Moreover, indole 3-Ethanol was found to be correlated with multiple genera, including *Eubacterium xylanophilum*, Oscillospiraceae UCG-003, Oscillospiraceae UCG−005, and Anaerovoracaceae Family XIII UCG−001.

### 3.3. VA Alters Serum Lipid Metabolic Profiles

**Figure 3:**
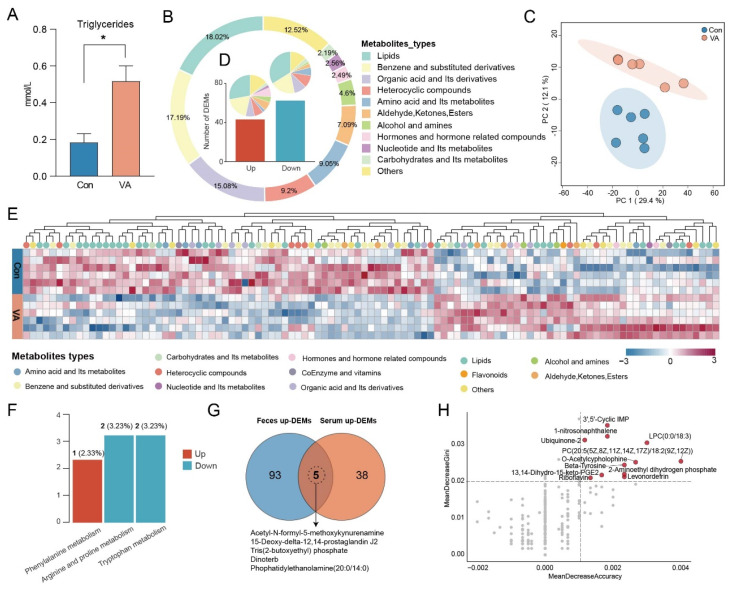
**Serum metabolite profiles and key metabolite identification in sika deer following vitamin A supplementation:** (**A**) Serum triglyceride concentration, * *p* < 0.05. (**B**) Pie chart of serum metabolite categories. (**C**) Principal component analysis of serum metabolite profiles. (**D**) Number and classification of up- and down-regulated differential metabolites. (**E**) Heatmap of differential serum metabolites. (**F**) KEGG pathway classification of differential serum metabolites. (**G**) Venn diagram showing overlapping upregulated metabolites between feces and serum. (**H**) Random forest analysis identifying key metabolites contributing to group discrimination.

We first measured the serum biochemical parameters and found that the triglyceride concentration was significantly elevated in the VA group compared to that in the Con group (*p* < 0.05, Figure 3A). Although the levels of high-density lipoprotein cholesterol, low-density lipoprotein cholesterol, and total cholesterol also exhibited an upward trend, the differences exhibited were not statistically significant (*p* > 0.05, Figure S1). Similarly, the serum VA concentration did not significantly differ between the two groups (Figure S1B).

To explore the systemic metabolic changes induced by VA supplementation, we performed untargeted metabolomic analyses of serum. A total of 1326 serum metabolites were identified and categorized into 13 categories, including lipids (18.02%); benzene and substituted derivatives (17.19%); organic acids and their derivatives (15.08%); heterocyclic compounds (12.52%); amino acids and their metabolites (9.2%); and aldehydes, ketones, and esters (7.09%) (Figure 3B). The PCA revealed a clear separation between the VA and Con groups (Figure 3C). Among the identified metabolites, 105 metabolites showed significant differences between the two groups, with 43 significantly increased and 62 decreased in the VA group compared to the Con group (Figure 3D). Notably, the increased metabolites were predominantly classified as lipids (e.g., nine phospholipids) and organic acids (e.g., 4-Acetamidobutanoic acid, Figure 3E). Enrichment analysis revealed that these increased metabolites were mainly enriched in phenylalanine metabolism, while decreased metabolites were enriched in arginine and proline metabolism and tryptophan metabolism (Figure 3F). Importantly, AMFK, 15-deoxy-12,14-prostaglandin J2(15d-PGJ2), Tris(2-butoxyethyl) phosphate, dinoterb, and phosphatidylethanolamine (20:0/14:0) were increased in both the feces and serum (Figure 3G). Furthermore, the random forest analysis further identified 11 key metabolites that contributed strongly to group discrimination, including 3′,5′-cyclic IMP, levonordefrin, β-tyrosine, and O-acetylcyphyllophine (Figure 3H).

### 3.4. Distinct Gene Expression of Antler Cartilage

**Figure 4:**
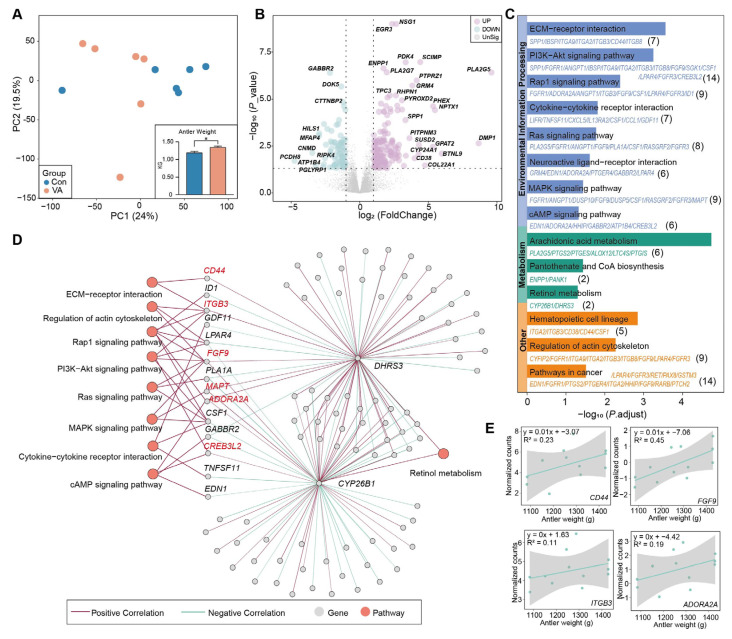
**Transcriptomic analysis of antler cartilage tissue and gene–pathway correlation network:** (**A**) Principal component analysis of cartilage transcriptome profiles; inset shows antler weight, * *p* < 0.05. (**B**) Volcano plot of differentially expressed genes. (**C**) KEGG pathway enrichment analysis of differentially expressed genes. (**D**) Correlation network linking differentially expressed genes and retinol metabolism-related genes with enriched pathways. (**E**) Correlations between gene expression levels of *CD44*, *ITGB3*, *FGF9*, and *ADORA2A* and antler weight.

We found that antler weight and antler average daily gain were significantly increased in the VA group compared to the Con group (Figure 4A and Figure S1C). To elucidate the molecular mechanisms underlying VA-induced antler growth, we performed transcriptomic profiling of the antler cartilage. The PCA revealed a distinct separation between the Con and VA groups (Figure 4A). A total of 241 DEGs were identified, including 156 upregulated (e.g., *DMP1*, *PLA2G5*, *SCIMP*, *PTPRZ1*, *NPTX1*, *BTNL9*, *COL22A1*, and *CYP24A1*) and 85 downregulated (e.g., *PCDH8*, *CNMD*, *ATP1B4*, *MFAP4*, *HILS*, and *GABBR2*) DEGs in the VA group (Figure 4B). The upregulated DEGs were enriched in 14 pathways, particularly those related to environmental information processing (e.g., ECM–receptor interaction, PI3K–Akt signaling, MAPK signaling, and cytokine–cytokine receptor interaction) and metabolism (e.g., retinol metabolism, arachidonic acid metabolism, and pantothenate and CoA biosynthesis) (Figure 4C). We identified six genes (*CD44*, *ITGB3*, *FGF9*, *MAPT*, *ADORA2A*, and *CREB3L2*) that were positively correlated with key regulators of the retinol metabolism pathway (*CYP26B1* and/or *DHRS3*) of retinol metabolism (Figure 4D). Notably, four genes (*CD44*, *ITGB3*, *FGF9*, and *ADORA2A*) were found to be positively correlated with the antler weight (Figure 4E). Furthermore, PPI analysis revealed a direct physical interaction between *FGF9* and *CYP26B1* (Figure S2A), further supporting its involvement in the retinoid-regulated cartilage signaling network.

### 3.5. AMFK-Linked Gene Module Associated with Antler Growth

**Figure 5:**
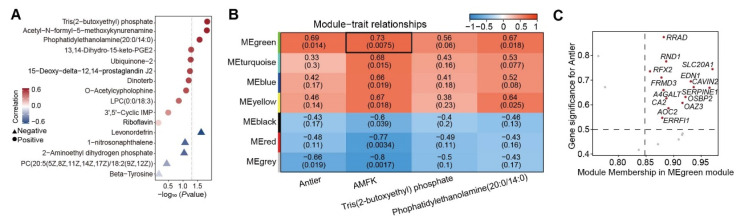
**Correlations of serum metabolites with antler weight and identification of key gene modules via WGCNA analysis:** (**A**) Correlations between selected serum metabolites and antler weight. (**B**) Module–trait relationship heatmap from weighted gene co-expression network analysis. (**C**) Scatter plot of gene significance for antler weight versus module membership in the MEgreen module.

Antlers are highly vascularized structures, suggesting that systemic metabolic processes play a critical role in supporting their rapid growth and development. To identify metabolites potentially involved in promoting antler growth, we analyzed the correlations between antler weight and five 5 metabolites shared by the feces and serum, as well as the 11 serum-specific metabolites. Among these, Tris(2-butoxyethyl) phosphate, AMFK, and phosphatidylethanolamine (20:0/14:0) showed significant positive correlations with the antler weight (Figure 5A). WGCNA of DEGs, antler weight, and the above metabolites identified the MEgreen module as being positively correlated with both antler weight and elevated metabolite levels in the VA group, including AMFK, 15d-PGJ2, and Tris(2-butoxyethyl) phosphate (Figure 5B). Notably, AMFK exhibited the strongest correlation (R = 0.73). Hub gene analysis within this module identified 14 genes, including *RRAD*, *RND1*, *SLC20A1*, *RFX2*, *FRMD3*, *EDN1*, *CAVIN2*, *SERPINE1*, *OSBP2*, *A4GALT*, *CA2*, *OAZ3*, *AOC2*, and *ERRFI1* (Figure 5C). To further explore the interactions among these 14 genes, we performed a PPI analysis focusing on *FGF9*, a gene previously identified as being strongly associated with both antler growth and retinol metabolism (Figure 4E). The results revealed that *EDN1* and *SERPINE1* directly interact with *FGF9* (Figure S2B), highlighting them as key candidate genes potentially mediating AMFK’s regulatory effects on chondrocyte activity and antler elongation.

### 3.6. AMFK Promotes Chondrocyte Proliferation

**Figure 6:**
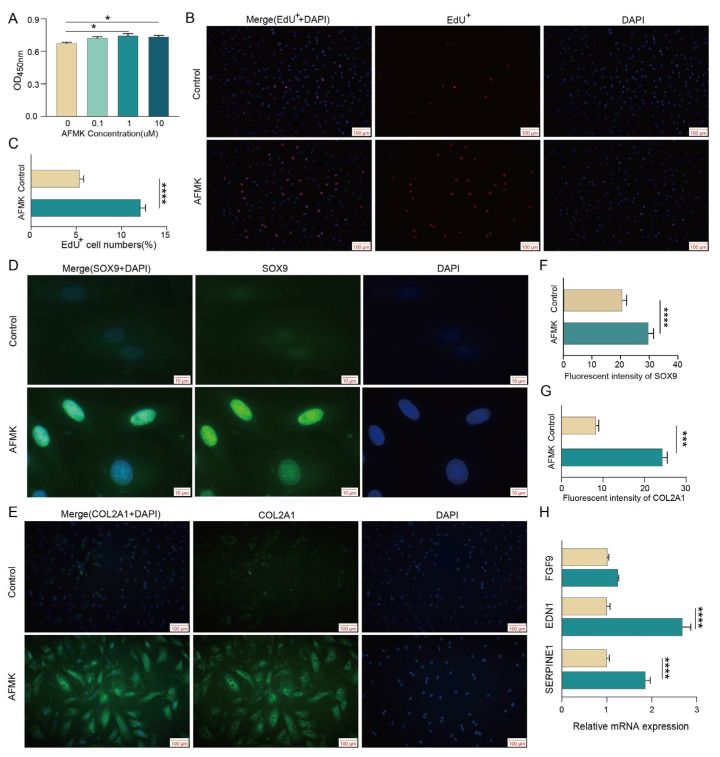
**Effects of AMFK on chondrocyte proliferation and chondrogenic marker expression in vitro**: (**A**) Cell proliferation analysis using the CCK-8 assay at different AMFK concentrations. (**B**) Representative images of EdU staining and DAPI counterstaining. (**C**) Quantification of EdU-positive cell percentage, **** *p* < 0.0001. (**D**,**E**) Immunofluorescence staining of SOX9 and COL2A1. (**F**,**G**) Fluorescence intensity quantification of SOX9 and COL2A1. (**H**) Relative mRNA expression levels of *FGF9*, *EDN1*, and *SERPINE1* were analyzed by qPCR., * *p* < 0.05, *** *p* < 0.001, **** *p* < 0.0001. Representative fields from independent biological replicates (n ≥ 3).

The CCK-8 assay results demonstrated that treatment with 1 μM and 10 μM AMFK significantly promoted the proliferation of antler chondrocytes, with the highest proliferation rate observed at 1 μM (Figure 6A). The EdU incorporation analysis further confirmed a significant increase in the proportion of EdU-positive cells at 1 μM treatment (Figure 6B,C), indicating enhanced DNA synthesis and cellular proliferative activity. Immunofluorescence staining showed that AMFK significantly increased the expression of *COL2A1* and *SOX9*, and SOX9 was highly concentrated in the nuclear region (Figure 6D–G), suggesting that AMFK facilitates extracellular matrix synthesis and supports the maintenance of the chondrogenic phenotype. Furthermore, the qPCR analysis revealed that AMFK significantly upregulates the expression of *SERPINE1* and *EDN1* mRNA (*p* < 0.05), while FGF9 exhibited an upward trend (Figure 6H). These results suggest that SERPINE1 and EDN1 may mediate AMFK’s proliferative effects on chondrocytes.

## 4. Discussion

The results of this study showed that dietary VA supplementation alters the fecal microbiota in sika deer, as evidenced by increased abundances of Oscillospiraceae, *Anaerovorax*, Rikenellaceae, and *Eubacterium xylanophilum*, which are associated with fatty acid metabolism, biotin biosynthesis, phosphonate and phosphinate metabolism, and glycosphingolipid biosynthesis. Consistent with these findings, previous studies have demonstrated that VA deficiency leads to significant disturbances in the gut microbial ecology, particularly characterized by a reduction in butyrate-producing bacteria and alterations in energy metabolism pathways [14]. The increased genera observed in the VA group have been associated with an enhanced fermentative capacity, primarily through fiber degradation [33]. Their enrichment suggests that VA promotes a gut microbial environment favorable for energy-efficient fermentation and contributes to the production of vitamin-derived cofactors [34,35]. Notably, VA-enriched taxa, such as Oscillospiraceae UCG-005 and *Anaerovorax*, exhibited significant correlations with several lipid mediators and anti-inflammatory compounds, including prostaglandin J2, palmitoylethanolamide, and epoprostenol. These findings are consistent with previous findings showing that VA regulates microbial metabolic activity under nutritional stress [14,36]. These results indicate that VA may modulate microbial composition and function to influence host lipid mediator production and inflammation resolution. We observed elevated TG levels and enriched phenylalanine metabolism in the VA group. Consistent with previous studies, VA was found to influence lipid homeostasis by regulating lipid synthesis, transport, and catabolism [14,37], and retinoids were confirmed to regulate lipid metabolism through the retinoid X receptor and peroxisome proliferator-activated receptor, which coordinate the transcription of genes involved in fatty acid metabolism, including triglyceride synthesis and lipid oxidation [38]. Moreover, VA deficiency has been shown to impair phenylalanine metabolism, which is essential for maintaining protein synthesis and vascular function [39,40,41]. Phenylalanine stimulated chondrocyte proliferation, thereby supplying essential structural and metabolic resources for rapid cartilage and bone development [42]. These findings suggest that VA supplementation modulates lipid and amino acid metabolism to support antler growth. Importantly, we also observed increased levels of hormones or hormone-related metabolites in the VA group, including prostaglandin J2, norepinephrine, and 11-dehydrocorticosterone. Previous studies have shown that gut microbial alterations can modulate systemic corticosteroid levels in mice [43]. Emerging evidence also indicates that melatonin can be influenced by the gut microbial composition and activity [44,45]. VA potentially regulates the biosynthesis of hormone-like metabolites, thereby contributing to systemic metabolic and endocrine homeostasis [14]. For instance, 15d-PGJ2 is an endogenous PPAR-γ ligand with well-characterized anti-inflammatory and remodeling actions; through PPAR-γ-dependent pathways, it can modulate gene programs linked to vascular and matrix regulation, providing a plausible mechanistic link to cartilage/antler biology [46], while norepinephrine enhances vascular perfusion and metabolic activation [47]. These findings suggest that VA may influence endocrine signaling by shaping the gut microbial community’s composition. Moreover, we identified that 3′,5′-cyclic IMP and AMFK as potential contributors to antler development. 3′,5′-cyclic IMP facilitates nucleotide metabolism and energy transfer, supporting rapid cell proliferation and matrix synthesis [48,49]. AMFK, a melatonin-derived metabolite, possesses antioxidant and cytoprotective properties [50]. A previous study has shown that melatonin promotes chondrocyte differentiation in sika deer [18]. Serum retinol did not differ between groups despite daily supplementation. This finding is consistent with the strong homeostatic regulation of circulating retinoids and with prior evidence in rats showing that local retinoid signaling can remain active even when systemic retinol is unchanged [51]. We therefore interpret the effects of vitamin A as predominantly local, mediated by microbiota-dependent metabolic shifts (e.g., AMFK) and tissue-level retinoid signaling in antler cartilage rather than by systemic increases in serum retinol. On the other hand, from a safety standpoint, the short-term rumen-protected VA dose (~40,000 IU/day) did not produce biochemical evidence of hepatotoxicity: serum ALT did not differ between groups, and serum VA in the VA group was only modestly higher than controls and not significant. Together with the absence of adverse clinical signs, these findings argue against systemic hypervitaminosis at the applied dose.

It has been demonstrated that VA promotes skeletal development [52] and the first antler growth [53]. In the present study, we found that the genes (*RDH13*, *SDR16*, *DHRS*, *ALDH1A*, and *CYP26B1*) involved in the conversion of all-trans-retinol to all-trans-retinoate, all-trans-4-hydroxyretinoic acid, and all-trans-4-oxoretinoic acid were upregulated in the VA group (Figure S3). This observation is consistent with previous findings reporting substantial levels of all-trans-4-oxoretinoic acid in antler tissue [7]. RA has been shown to directly regulate antler regeneration by modulating cell differentiation during the process of endochondral ossification [7]. Endogenous retinoids and the enzymes responsible for RA biosynthesis have been identified in critical regions of antler tissue, including the epidermis, perichondrium, and mesenchymal zones near the growing tip [7]. Moreover, we found that genes involved in retinol metabolism were enriched in the VA group. These results suggested that VA supplementation may enhance local RA signaling in antler tissue, indicating that local RA metabolism signaling is active during growth. Several genes associated with bone and cartilage development, including *DMP1*, *COL22A1*, and *PLA2G5*, were significantly upregulated in the VA group. *DMP1* is a highly phosphorylated extracellular matrix protein predominantly expressed in mineralized tissues and is known to be upregulated during bone repair [54]. *COL22A1*, identified as a structural ECM component in decellularized cartilage matrices, has been suggested to influence the adhesive microenvironment of chondrocytes and support cells’ proliferative potential [55]. These findings suggest that the involvement of enhanced extracellular matrix remodeling and chondrocyte proliferation is involved in supporting the rapid cartilage and bone development observed during antler growth. Further linear regression analyses indicated that *CD44*, *FGF9*, *ITGB3*, and *ADORA2A* were positively associated with antler weight. CD44 is a key cell surface receptor involved in hyaluronan binding and extracellular matrix interactions, playing crucial roles in chondrocyte proliferation, migration, and cartilage homeostasis [56]. FGF9, a member of the fibroblast growth factor family that promotes chondrocyte proliferation and differentiation and has been implicated in endochondral ossification and skeletal development [57]. ITGB3 encodes integrin β3, a subunit involved in cell adhesion and signaling, facilitating chondrocyte attachment to the extracellular matrix during cartilage remodeling [58]. Collectively, these findings suggest that these genes may contribute to antler cartilage growth by enhancing chondrocyte proliferation, improving matrix interactions, and facilitating extracellular matrix remodeling, ultimately leading to increased antler weight.

The WGCNA highlighted AMFK as a metabolite associated with antler weight. AMFK arises from melatonin catabolism and is influenced by tryptophan availability and microbial metabolism [50]. Vitamin A has been reported to affect melatonin by modulating retinal and pineal retinoid signaling and by shaping the gut microbiota, which together influence tryptophan-derived serotonin and melatonin synthesis [17,59]. Accordingly, the observed increase in AMFK should be interpreted as VA-associated rather than VA-specific. In our data, AMFK was attributed primarily to host-microbe co-metabolism and correlated with cartilage gene programs; we therefore regard AMFK as a VA-associated, microbiota-influenced candidate mediator within the host-microbiota axis. Consistent with this view, AMFK significantly promoted chondrocyte proliferation and upregulated *SOX9* and *COL2A1* expression, suggesting that AMFK enhances both the proliferation and maturation of chondrocytes [60,61]. *SERPINE1* encodes a serine protease inhibitor involved in extracellular matrix remodeling and cellular migration [62], while *EDN1* is known to promote angiogenesis and support nutrient supply in proliferative tissues [63]. Moreover, melatonin has been widely recognized for its role in promoting chondrocyte proliferation and enhancing extracellular matrix synthesis [17,18]. These results suggest that AMFK, as a melatonin-derived metabolite, may play a role in contributing to cartilage development during antler growth. Based on published human and rodent data, AMFK in systemic circulation is typically at low picomolar or even undetectable levels, whereas local tissues or inflammatory contexts can reach the nanomolar range [64,65,66]. The 0.1–10 μM range used in vitro should therefore be viewed as pharmacologic and intended for mechanistic probing rather than a direct mimic of circulating exposure, and we plan targeted LC–MS/MS quantification of AMFK in deer serum and antler tissues in future work to anchor in vitro dosing to in vivo exposure.

The study is limited by the small sample size (*n* = 6 per group), the absence of a dose–response design, and the lack of orthogonal validation (e.g., qPCR for selected DEGs and targeted AMFK assays). Because only a single VA dose was tested, we cannot characterize dose–response relationships, identify minimal effective or upper safe levels, or evaluate potential nonlinearity or threshold effects; the current findings therefore pertain to the tested dose only. We also did not measure physiological stress biomarkers (e.g., cortisol), so residual handling- or housing-related stress effects cannot be excluded. Multi-omics integration can risk over-interpretation; we mitigated this by prespecifying selection criteria and controlling the false discovery rate, but confirmation in larger cohorts is needed. Additionally, targeted LC–MS/MS quantification and causal tests (e.g., VA dose–response, microbiota modulation, and isotope tracing along the tryptophan-melatonin-AMFK axis) are required to establish specificity and mediation. Future studies will implement multi-dose regimens with pharmacokinetic and targeted metabolite measurements to map exposure-response curves and establish causal thresholds.

## 5. Conclusions

In summary, VA supplementation altered the fecal microbiota community, promoting the proliferation of taxa associated with enhanced fermentative capacity. These microbial alterations contributed to the enrichment of bioactive metabolites, including the melatonin-derived signaling molecule AMFK, in both the gut and serum. Integrated multi-omics analysis revealed that AMFK was strongly associated with increased antler weight and the expression of genes involved in antler cartilage development. In vitro assays further demonstrated that AMFK promotes chondrocyte proliferation and supports the chondrogenic phenotype, accompanied by upregulation of *SERPINE1* and *EDN1* expression. Collectively, these findings demonstrate that VA modulates gut microbiota and metabolic profiles to promote cartilage proliferation and antler growth in sika deer. Given the small sample and single-dose design, these findings should be interpreted cautiously and verified in larger, multi-dose studies. Future work will implement multi-dose regimens with targeted LC–MS/MS quantification of AMFK and retinoids in serum and antler tissues, microbiota modulation, and isotope tracing along the tryptophan–melatonin–AMFK axis, and mechanistic validation to establish specificity, causality, and exposure–response relationships.

## Data Availability

The datasets generated in this study are publicly available in the NCBI repository under the accession numbers PRJNA1291056 and PRJNA1291498.

## Supplementary Materials

The following supporting information can be downloaded at: https://www.mdpi.com/article/10.3390/ani15192879/s1, Supplementary Figure S1:Serum biochemical parameters and vitamin A levels in sika deer: (A) Serum levels of cholesterol (CHO), low-density lipoprotein cholesterol (LDL-C), high-density lipoprotein cholesterol (HDL-C), total protein (TP), alkaline phosphatase (ALP), alanine aminotransferase (ALT), and albumin (ALB) in control and VA-supplemented groups. (B) Serum concentration of VA. Supplementary Figure S2: Protein–protein interaction networks related to antler growth: (A) Interaction network of FGF9 with vitamin A-responsive genes identified in antler tissue transcriptome. (B) Interaction network of FGF9 with AMFK-associated hub genes identified from the MEgreen module in WGCNA. Known and predicted interactions were visualized using the STRING database (v12.0). Edge colors represent different evidence sources, including experimental determination, gene neighborhood, co-expression, and text mining. Supplementary Figure S3: Differentially expressed genes involved in retinol metabolism in sika deer antler cartilage. Pathway map of retinol metabolism generated from the KEGG database. Genes outlined in red were significantly upregulated (*p* < 0.05, log_2_FC > 0) in the VA group compared to the control group based on transcriptomic analysis of antler cartilage. Supplementary Table S1: Dietary composition and nutrient levels. Supplementary Table S2: The primer sequences. Supplementary Table S3: 106 DEM in feces. Supplementary Table S4: Metabolites-Origin of 106 DEM.
