# Supplementary material for: Vitamin A Supplementation Induces AMFK Production to Promote Cartilage Proliferation and Antler Growth in Sika Deer
Source: Animals (Basel). 2025 Oct 1;15(19):2879. doi: 10.3390/ani15192879 (PMC12523666; doi:10.3390/ani15192879)
Supplement: Supplementary file 1 [file animals-15-02879-s001.zip › Supplementary table 2.pdf]

Table S2. Primer sequences

Gene name	Forward	Reverse
FGF9	*GTATAGCCGTGGGTCTGGTCAG*	*CGTAGAACCTCCGTCCAGTGTC*
EDN1	*CTGCTCCTGCTCCTCCTTGAT*	*GCACACTGGCATCTCTTCCTG*
SERRINE	*CCAAGAGCACCGTCCAGAGA*	*GGAGGCAGACCCTTCACCAAA*
GAPDH	*AGATGGTGAAGGTCGGAGTG*	*CCTTTCCATTGATGACGAGC*
