# Supplementary material for: Vitamin A Supplementation Induces AMFK Production to Promote Cartilage Proliferation and Antler Growth in Sika Deer
Source: Animals (Basel). 2025 Oct 1;15(19):2879. doi: 10.3390/ani15192879 (PMC12523666; doi:10.3390/ani15192879)
Supplement: Supplementary file 1 [file animals-15-02879-s001.zip › Supplementary table 1.pdf]

**Table S1**. Basal diet

| Ingredient, (g/100 g) DM | | Nutrient composition, % DM | |
|---|---|---|---|
| Corn silage | 30.0 | Crude protein | 16.25 |
| Alfalfa | 15.0 | Neutral detergent fiber | 59.54 |
| Corn grain | 15.0 | Acid detergent fiber | 34.68 |
| Soybean meal | 20.0 | ME, MJ/kg | 11.24 |
| DDGS | 5.0 | | |
| Corn germ | 8.0 | | |
| Corn fiber | 5.0 | | |
| NaCl | 1.0 | | |
| Premix[1] | 1.0 | | |
| Total | 100.00 | | |

1. Formulated to provide (per kg of DM): 150 g of salt, 200 g of $NaHCO_3$, 75 g of Ca, 20 g of P, 600 mg of Mn, 680 mg of Fe, 960 mg of Zn, 300 mg of Cu, 140,000 IU of vitamin A, 55,000 of vitamin $D_3$, 700 IU of vitamin E, and 600 mg of niacin.
