# Supplementary material for: Vitamin A Supplementation Induces AMFK Production to Promote Cartilage Proliferation and Antler Growth in Sika Deer
Source: Animals (Basel). 2025 Oct 1;15(19):2879. doi: 10.3390/ani15192879 (PMC12523666; doi:10.3390/ani15192879)
Supplement: Supplementary file 1 [file animals-15-02879-s001.zip › S2.pdf]

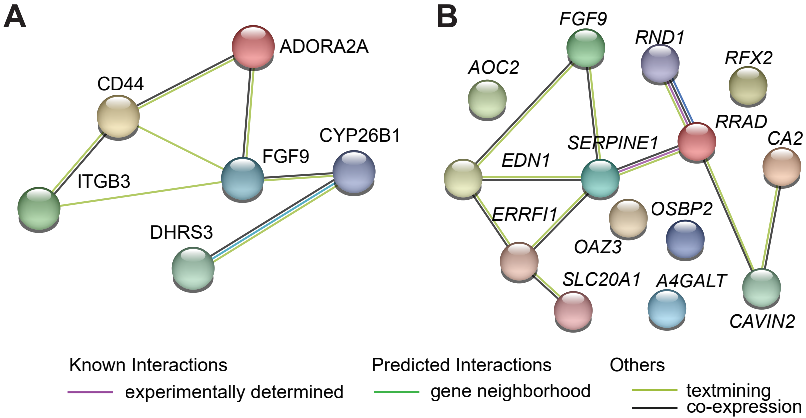
A
ADORA2A
CD44
CYP26B1
FGF9
ITGB3
DHRS3
B
FGF9
RND1
AOC2
RFX2
RRAD
CA2
SERPINE1
EDN1
ERRFI1
OSBP2
OAZ3
SLC20A1
A4GALT
CAVIN2
Known Interactions
experimentally determined
Predicted Interactions
gene neighborhood
Others
textmining
co-expression
