# Supplementary figures and images for: Vitamin A Supplementation Induces AMFK Production to Promote Cartilage Proliferation and Antler Growth in Sika Deer
Source: Animals (Basel). 2025 Oct 1;15(19):2879. doi: 10.3390/ani15192879 (PMC12523666; doi:10.3390/ani15192879)

A

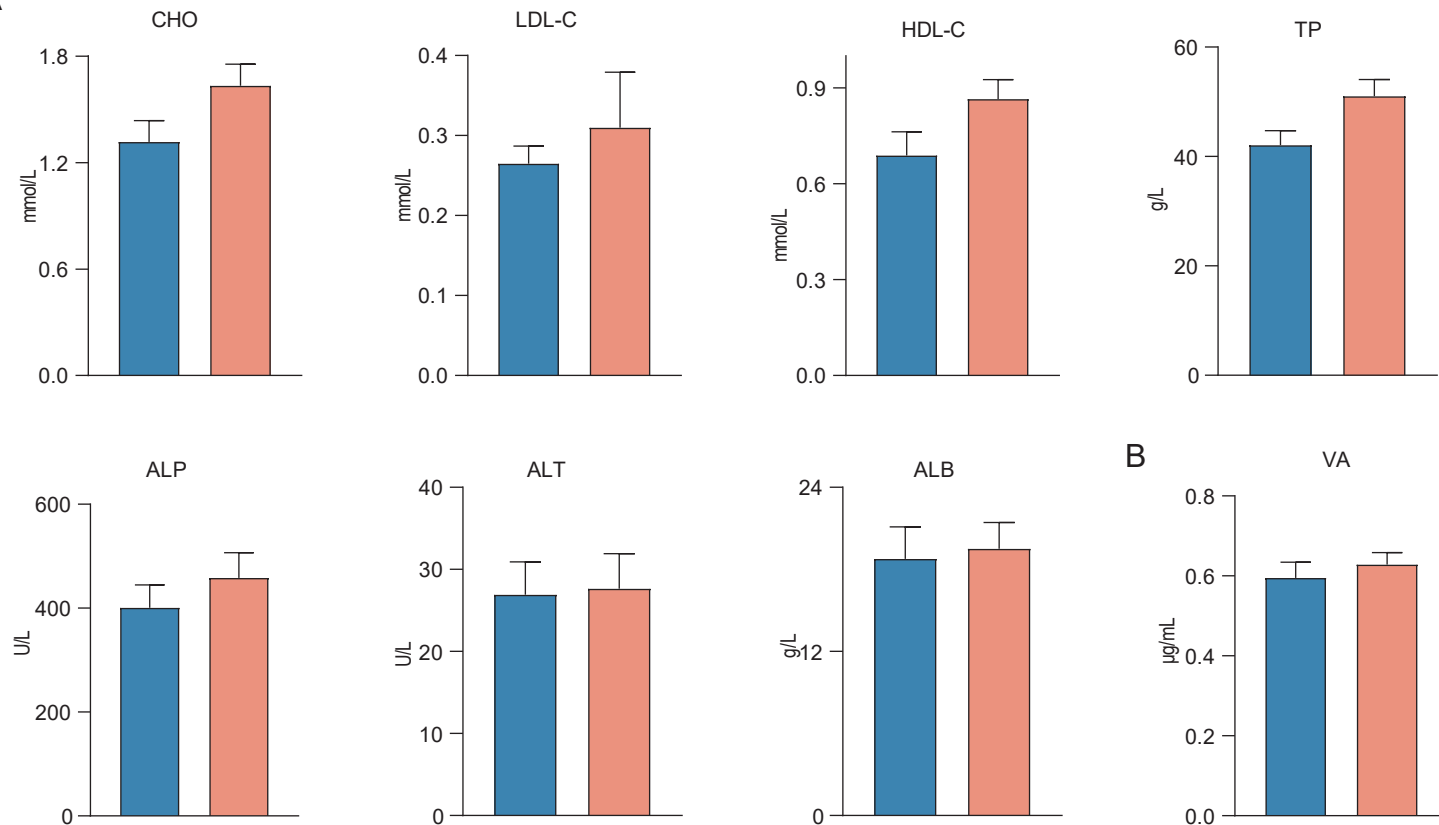

B

Supplement: Supplementary file 1 [file animals-15-02879-s001.zip › S1.pdf]

# Retinol Metabolism

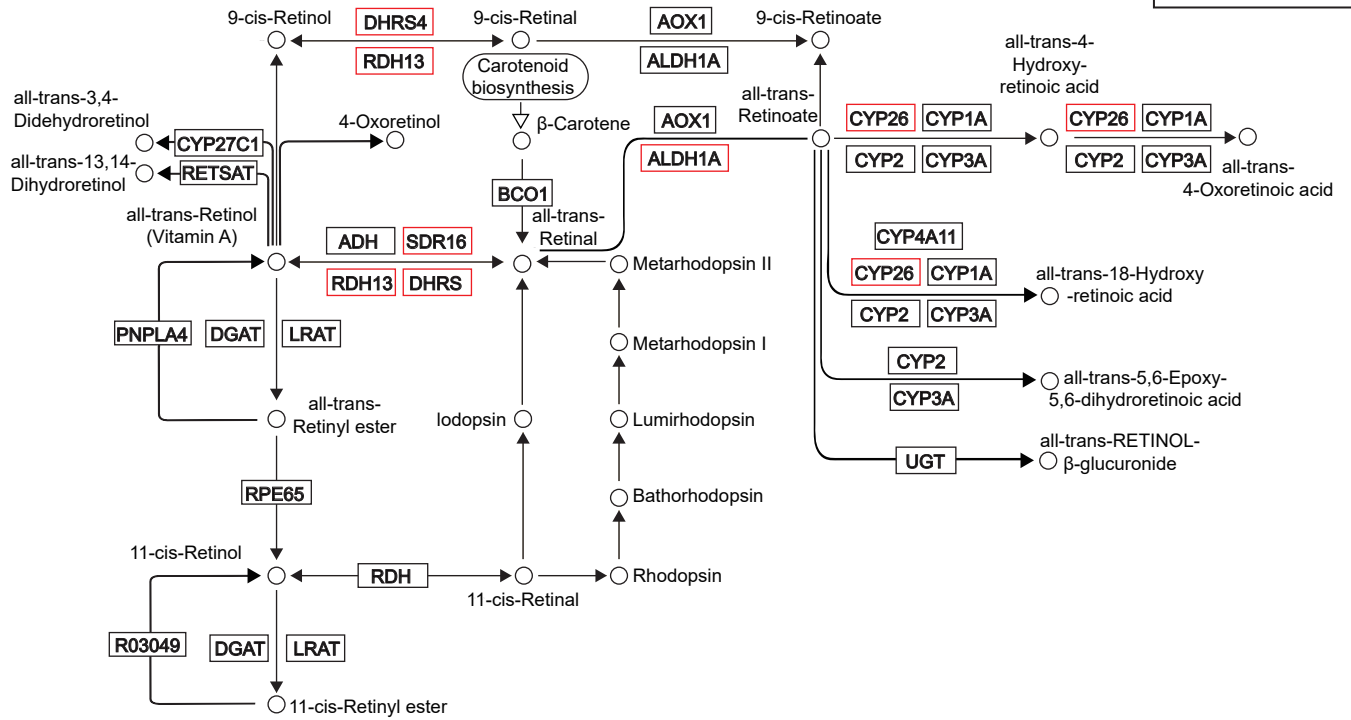

Supplement: Supplementary file 1 [file animals-15-02879-s001.zip › S3..pdf]
